# Evidence-based Treatment of Failed Primary Osteochondral Lesions of the Talus: A Systematic Review on Clinical Outcomes of Bone Marrow Stimulation

**DOI:** 10.1177/1947603521996023

**Published:** 2021-02-22

**Authors:** Jari Dahmen, Eoghan T. Hurley, Yoshiharu Shimozono, Christopher D. Murawski, Sjoerd A. S. Stufkens, Gino M. M. J. Kerkhoffs, John G. Kennedy

**Affiliations:** 1 Amsterdam UMC, University of Amsterdam, Department of Orthopaedic Surgery, Amsterdam Movement Sciences, Amsterdam, the Netherlands; 2 Academic Center for Evidence-based Sports Medicine (ACES), Amsterdam, the Netherlands; 3 Amsterdam Collaboration for Health and Safety in Sports (ACHSS), International Olympic Committee (IOC) Research Center Amsterdam UMC, Amsterdam, the Netherlands; 4 Department of Orthopaedic Surgery, NYU Langone Health, New York, NY, USA; 5 Department of Orthopaedic Surgery, Royal College of Surgeons in Ireland, Dublin, Ireland; 6 Department of Orthopaedic Surgery, Kyoto University Graduate School of Medicine, Kyoto, Japan; 7 Department of Orthopaedic Surgery, University of Pittsburgh School of Medicine, Pittsburgh, PA, USA

**Keywords:** osteochondral lesions, talus, bone marrow stimulation, microfracture, revision, nonprimary, arthroscopy, failed surgery

## Abstract

**Objective:**

The purpose of this study is to systematically review the literature and to evaluate the outcomes following bone marrow stimulation (BMS) for nonprimary osteochondral lesions of the talus (OLT).

**Design:**

A literature search was performed to identify studies published using PubMed (MEDLINE), EMBASE, CDSR, DARE, and CENTRAL. The review was performed according to the PRISMA guidelines. Two authors separately and independently screened the search results and conducted the quality assessment using the Methodological Index for Non-Randomized Studies (MINORS). Studies were pooled on clinical, sports, work, and imaging outcomes, as well as revision rates and complications. The primary outcome was clinical success rate.

**Results:**

Five studies with 70 patients were included in whom nonprimary OLTs were treated with secondary BMS. The pooled clinical success rate was 61% (95% confidence interval [CI], 50-72). The rate of return to any level of sport was 83% (95% CI, 70-91), while the return to pre-injury level of sport was 55% (95% CI, 34-74). The rate of return to work was 92% (95% CI, 78-97), and the complication rate was assessed to be 10% (95% CI, 4-22). Imaging outcomes were heterogeneous in outcome assessment, though a depressed subchondral bone plate was observed in 91% of the patients. The revision rate was 27% (95% CI, 18-40).

**Conclusions:**

The overall success rate of arthroscopic BMS for nonprimary osteochondral lesions of the talus was 61%, including a revision rate of 27%. Return to sports, work, and complication outcomes yielded fair to good results.

## Introduction

Osteochondral lesions of the talus (OLT) are a common injury in athletes and often result from acute ankle sprains, fractures, or chronic ankle instability. The initial nonoperative management of an OLT consists of physical therapy, immobilization, and the usage of nonsteroidal anti-inflammatory drugs or other painkillers.^1-3^ Once conservative treatment fails, surgical treatment, including bone marrow stimulation (BMS) for primary lesions up to 15 mm in diameter, can be considered.^4-9^ The ideal sized lesion treated with BMS is considered to be up to 10 mm in diameter and results in the formation of a blood marrow filling of the defect, later differentiating in bone and fibrocartilage on top, and this treatment can produce good results in the majority of patients at short- and midterm follow-up.^9-11^ However, there is concern that the fibrocartilage and the associated clinical outcomes may deteriorate over time and require revision treatment, thereby yielding worse outcomes at longer term follow-up.^9,12,13^

When examining the evidence, it is clear that the majority of the literature focuses on BMS for primary (i.e., osteochondral lesions that have not had prior surgery) OLTs.^1,14-16^ A recent systematic review on primary lesions by Dahmen *et al*.^1^ found that 82% of the primary OLTs treated with BMS resulted in successful outcomes. It is assumed that BMS for nonprimary lesions (i.e., osteochondral lesions that have had prior surgical intervention(s)) results in worse outcomes in comparison to primary arthroscopic treatment; however, there is a clear paucity of clinical data and thus limited knowledge on the efficacy of BMS for nonprimary OLTs.^6,17-19^ The purpose of this study is to systematically review the literature and to evaluate the outcomes following BMS for nonprimary OLTs. The hypothesis was that BMS in revision surgery would result in better outcomes than primary treatment due to the centralization of more complex cases.^2^

## Materials and Methods

The systematic review was prospectively registered at the PROSPERO register under number CRD42018082150.^20^

### Search Strategy

A systematic review was performed by 2 independent blinded reviewers (JD and EH) according to the Preferred Reporting Items for Systematic Reviews and Meta-Analyses (PRISMA) guidelines. The MEDLINE, EMBASE, and The Cochrane Library databases were searched from January 1996. This time frame was chosen by the authors as the arthroscopic techniques for treating OLT were developed and established by 1996 in the orthopedic field.^21^ The search algorithm that was used for the search is presented in the appendix. Backward citation chaining strategy was applied as an additional search technique.

### Eligibility Criteria and Study Selection

The inclusion and exclusion criteria of the present study are presented in **Table 1**. The titles and abstracts were screened by 2 reviewers (JD and EH) using the inclusion and exclusion criteria. Subsequently, full texts of potentially relevant studies were then reviewed. The references of all of the studies receiving full-text review were screened for additional articles that were not identified through our search strategy. Studies were included for further analysis with the agreement of both independent reviewers; instances of disagreement were settled in consultation with a third author (GK). When necessary, authors were contacted to ask the nature of the defect (primary or nonprimary) and to separate data on solely nonprimary lesions. When no reply was reported, contact was sought via 2 reminder e-mails. If no response was recorded, the article in question was excluded. Studies were not blinded for author, affiliation, or source.

**Table 1.** Inclusion and Exclusion Criteria.

Inclusion Criteria	Exclusion Criteria
Clinical studies reporting outcomes of arthroscopic BMS for nonprimary talar OCLs	Data not interpretable
Levels I-IV clinical studies	Combination of patient/treatment groups and/or no separate data per group
Five or more patients included	Follow-up period <6 months
Peer-reviewed studies	Patient overlap in different studies
Full-text available studies published in the English language	Treatment option inappropriately described
	Asymptomatic lesions
	Level V evidence
	Animal studies/cadaveric studies

BMS = bone marrow stimulation; OCL = osteochondral lesion.

### Assessment of Level and Quality of Evidence

The level of evidence of the included studies was evaluated based on the criteria from the Oxford Centre for Evidence-based Medicine. The methodological quality of evidence was evaluated by 2 independent investigators (JD and EH) using the METHODOLOGICAL index for Nonrandomized Studies (MINORS) tool.^22^ The MINORS tool consists of 8 nonrandomized or 12 items for comparative nonrandomized studies. Maximum scores are 16 for noncomparative nonrandomized studies and 26 for comparative nonrandomized studies. Instances of discrepancy were resolved by consensus, and if any disagreement persisted, a senior author (GK) was consulted and a consensus was reached.

### Data Extraction

The data of each study were extracted using a standardized data sheet consisting of the predetermined list of information required. All available data on patient characteristics were retrieved due to the scarcity of the literature on the particular topic. Preoperative and postoperative clinical outcome scores were extracted on mean scores, subjective satisfaction, and number of patients treated successfully. Preoperative and postoperative imaging outcomes were also included in the study. All clinical and sports outcomes reported were evaluated. Wherever possible, the number of successfully treated lesions were also assessed. The surgical intervention was defined as successful when a good or excellent result at follow-up was reported with any accepted associated clinical scoring system (American Orthopaedic Foot and Ankle Score [AOFAS] at or above 80; Foot and Ankle Ability Measure (FAAM) at or above 80; or any other clinical scoring system rating the outcome as good or excellent post-operatively).^23,24^ Mean postoperative clinical scores were pooled whenever possible.

### Statistical and Data Analysis

Weighted means and ranges of original data were used in case of descriptive values. Success rates as well as return to sports and work rates were calculated per study with a 95% binomial proportion confidence interval, and—whenever possible—pooled success rates were calculated with the Wilson score interval (CIA; Confidence Interval Analysis for Windows, version 2.2.0).^25^ The overall pooled clinical success rate was regarded as the primary outcome for the present study. All other outcomes were classified as secondary outcomes. The remaining statistical analyses were performed using Microsoft Excel and SPSS (IBM Corp., Released 2013, IBM SPSS Statistics for Macintosh, Version 22.0, Armonk, NY).

## Results

**Figure 1.**
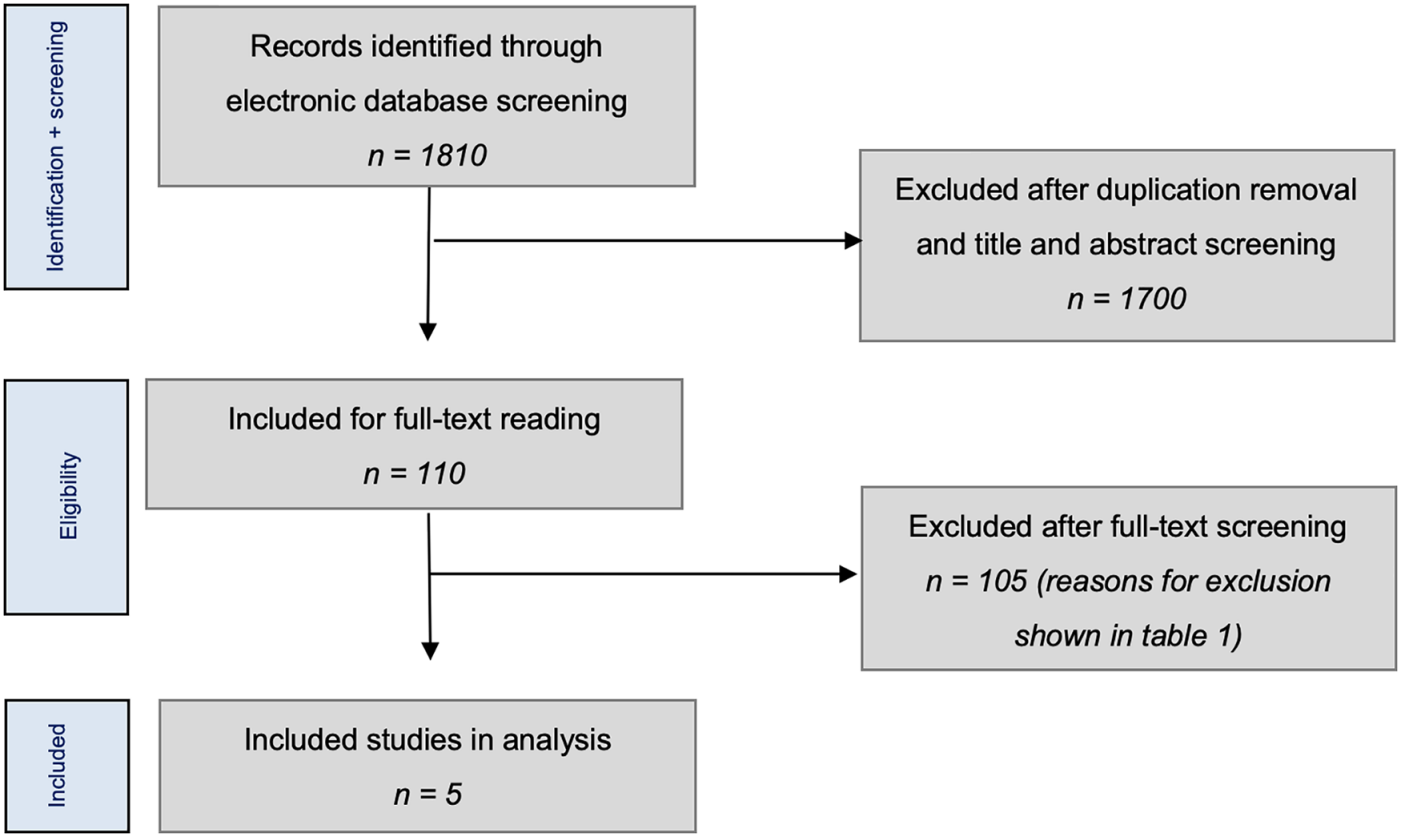
Literature selection algorithms: Preferred Reporting Items for Systematic Reviews and Meta-Analyses (PRISMA).

Overall, 1810 studies were initially identified from the literature. No additional studies were added through reference and/or citation searches. In total, 11 author groups were contacted to request data according to our inclusion and exclusion criteria. After application of inclusion and exclusion criteria, 5 studies reporting 70 ankles were included in the final analysis^19,26-29^ (**Fig. 1**).

### Evaluation of the Characteristics of Included Studies

A total of 70 patients were included; the average age was 33 years, and 63% were male. A history of trauma was reported in 65% of cases, and further characteristics are shown in **Table 2**. The procedure before the nonprimary BMS was noted as removal of an osteochondral fragment in 21 cases and open or arthroscopic BMS in 50 cases. The time between the previous surgery and current arthroscopic surgery was reported in 3 studies,^19,26,27^ and the weighed mean was 28 months (8-108). The mean weighted follow-up was 49 months (13-71).

**Table 2.** Study characteristics and patient demographic characteristics.

Authors, Year	Patients, *n*	Prospective or Retrospective	Treatment	LOE	MQOE	Male/Female	Side, R/L	Mean Age, Years [Range]	Mean Follow-up, Months [Range]	Mean Preoperative Size, mm^2^/mm^3^ [Range]	Location	Preoperative Radiology (System) Used
Ogilvie-Harris and Sarrosa,^26^ 1999	8	Retrospective	Arthroscopic debridement	IV	3/16	4/4	4/4	33 [22-42]	38 [24-63]	NA	4 PM, 4 AL	Radiographs (Berndt and Harty)
Reilingh *et al*.,^27^ 2016	12	Prospective	Arthroscopic microfracture	I	21/14	8/4	7/5	32 [20-41]	13 [12-20]	102 mm^2^ [60-196]/782mm^3^ [360-1764]	1 AM, 2 CM, 3 PM, 1 AL, 2 CL, 1 PL, 1 AC, 1 CC	CT (Berndt and Harty)
Savva *et al*.,^19^ 2007	12	Retrospective	Arthroscopic debridement	IV	9/16	7/5	NA	31 [20-41]	71 [18-133]	72 mm^2^ [25-120]/NA	6 medial, 6 lateral	Radiographs and MRI (Berndt and Harty)
Schuman *et al*.,^28^ 2002	16	Prospective	Arthroscopic debridement and drilling	IV	7/16	7/9	NA	24 [16-38]	64 [29-129]	NA/NA	12 medial, 4 lateral	Radiographs and CT (Berndt and Harty)
Yoon *et al*.,^29^ 2014	22	Retrospective	Arthroscopic microfracture or abrasion arthroplasty	III	20/24	18/4	NA	42 [NR]	50 [24-116]	139 mm^2^ [NR]/NR	6 anterior, 8 central, 8 posterior of which 18 medial and 4 lateral	Radiographs and MRI (Berndt and Harty)

LOE = level of evidence; MQOE = methodologic quality of evidence; NA = not available; AC = anterocentral; CM = centromedial; AM = anteromedial; PM = posteromedial; CL = centrolateral; PL = posterolateral; AL = anterolateral; CC = centrocentral; CT = computed tomography; MRI = magnetic resonance imaging.

### Nature of Radiological Lesion Morphology, Concomitant Procedures/Lesions, and Adjunct Procedures

In 2 studies, information on the presence or absence of preoperative cysts were not present. One study excluded lesions with cysts, while in the group studied by Reilingh *et al*.,^27^ there was solely one cyst included of the 12 (8%), and in the study by Yoon *et al*.,^29^ 14 out of the 22 (64%) patients had preoperative cyst morphology. The study of Yoon *et al*.^29^ mentioned whether lesions had uncontained or contained nature, of which 3 (14%) were contained lesions and 19 (86%) were uncontained.

Concerning concomitant procedures performed or concomitant diagnoses mentioned, Savva *et al*.^19^ revealed that in the index procedure 3 of 12 patients received a stabilization procedure for concomitant ankle instability and one of the patients received an arthroscopic debridement in the ankle. Schuman *et al*.^28^ and Ogilvie-Harris and Sarrosa^26^ did not mention whether there were patients included with concomitant lesions or with concomitant procedures having been performed. By comparison, Yoon *et al*.^29^ only included patients without ankle instability but did include 3 patients with tibial chondral lesions, 14 patients with soft-tissue impingement, and 10 patients with loose bodies. Reilingh *et al*.^27^ excluded patients with concomitant lower extremity diseases.

The study by Reilingh *et al*.^27^ included 2 study groups—an interventional group and a control group—studying the addition of hyaluronic acid to arthroscopic BMS. Due to the fact that no differences were found in clinical outcomes, the secondary OLT patients from both groups were merged. The other studies did not include any additional (biological) adjuncts.

### Methodological Quality

Three studies were retrospective, and 2 studies were prospective. There were 2 comparative studies and 3 noncomparative studies. The average MINORS score of the noncomparative studies was 6.3 (range, 3-9) out of a possible 16 points. The 2 comparative studies had an average MINORS score of 20.5 (range, 20-21) out of a possible 24 points.

### Clinical Outcomes

The mean success rates ranged from 32% to 88% (95% confidence interval [CI], 16-98), and the overall pooled success rate was 61% (95% CI, 50-72). The AOFAS was the most frequently used clinical scoring system,^19,27,29^ and the weighted mean improved from 50 (42-58) preoperatively to 76 (70-81) postoperatively. All mean preoperative and postoperative clinical scores can be found in **Tables 3** and **4**.

**Table 3.** Preoperative Clinical Scores.

Authors, Year	Preoperative Ogilvie-Harris	Preoperative AOFAS [Range]	Preoperative VAS/NRS [Range]	Preoperative FAOS [Range]	Preoperative EQ-5D [Range]	Preoperative AAS [Range]	Preoperative Tegner Score [Range]
Ogilvie-Harris and Sarrosa,^26^ 1999	Pain: 3 poor, 4 fair, 1 good Swelling: 1 poor, 4 fair, 3 good Stiffness: 0 poor, 4 fair, 4 good Limp: 1 poor, 5 fair, 2 good Activity: 2 poor, 5 fair, 1 good	NA	NA	NA	NA	NA	NA
Reilingh *et al*.,^27^ 2016	NA	58 [42-72]	Rest: 3.3 [0-6] Running: 8.0 [4-10]	Symptoms: 61 [14-89] Pain: 56 [31-89] ADL: 65 [44-97] Sport: 37 [15-90] QoL: 26 [0-56]	0.6 [0.2-0.9]	6.8 [4-9]	NA
Savva *et al*.,^19^ 2007	NA	42 [28-67]	NA	NA	NA	NA	NA
Schuman *et al*.,^28^ 2002	Pain: 7 poor, 8 fair, 1 good, 0 excellent Swelling: 2 poor, 7 fair, 0 good, 7 excellent Stiffness: 0 poor, 6 fair, 5 good, 5 excellent Limp: 2 poor, 4 fair, 2 good, 8 excellent Activity: 5 poor, 11 fair, 0 good, 0 excellent	NA	NA	NA	NA	NA	2.7 [NA]
Yoon *et al*.,^29^ 2014	NA	50 [NA]	General VAS: 6 [NA]	NA	NA	NA	NA

NA = not available; AOFAS = American Orthopaedic Foot and Ankle Score; VAS = Visual Analogue Scale; NRS = Numeric Rating Scale; FAOS = Foot and Ankle Outcome Score; ADL = activities of daily living; QoL = quality of life; EQ-5D = EuroQol; AAS = Ankle Activity Scale (values are presented in means).

**Table 4.** Postoperative Clinical Scores.

Authors, Year	Postoperative Ogilvie-Harris	Postoperative AOFAS [Range]	Postoperative VAS/NRS [Range]	Postoperative FAOS [Range]	Postoperative EQ-5D [Range]	Postoperative Satisfaction	Postoperative AAS [Range]	Postoperative Tegner Score [Range]	RTW Rate [95% CI] and Time in Weeks [Range]	RTS Rate [95% CI] and Time in Weeks [Range]	Success Rate [95% CI]
Ogilvie-Harris and Sarrosa,^26^ 1999	Pain: 0 poor, 1 fair, 4 good, 3 excellent Swelling: 0 poor, 1 fair, 4 good, 3 excellent Stiffness: 0 poor, 1 fair, 2 good, 5 excellent Limp: 0 poor, 1 fair, 4 good, 3 excellent Activity: 0 poor, 1 fair, 2 good, 5 excellent	NA	NA	NA	NA	NA	NA	NA	100% [67-100] Time: 6 weeks [2-14]	Any level: 88% [55-98] Pre-injury level: 38% [14-69] Time: NA	88% [53-98]
Reilingh *et al*.,^27^ 2016	NA	81 [43-100]	Rest: 1.1 [0-4] Running: 3.4 [0-7] Satisfaction: 6 [0-10]	Symptoms: 66 [32-100] Pain: 80 [50-92] ADL: 89 [69-100] Sport: 58 [0-100] QoL: 50 [6-81]	0.8 [0.3-1]	See NRS	6 [4-9]	NA	100% [76-100] Time: 8 weeks [2-13]	Any level: 83% [55-95] Pre-injury level: NA Time: 19 weeks [5-49]	75% [47-91]
Savva *et al*.,^19^ 2007	NA	81 [36-95]	NA	NA	NA	11 satisfied, 1 dissatisfied	NA	NA	NA	Any level: 92% [65-99] Pre-injury level: 67% [39-86] Time: NA	67% [39-86]
Schuman *et al*.,^28^ 2002	Pain: 2 poor, 1 fair, 7 good, 6 excellent Swelling: 1 poor, 2 fair, 4 good, 9 excellent Stiffness: 2 poor, 2 fair, 7 good, 5 excellent Limp: 1 poor, 2 fair, 3 good, 10 excellent Activity: 2 poor, 1 fair, 5 good, 8 excellent	NA	NA	NA	NA	NA	NA	5.6 [NA]	81% [57-93] Time: NA	Any level: 75% [51-90] Pre-injury level: NA Time: NA	75% [51-90]
Yoon *et al*.,^29^ 2014	NA	70 [NA]	5 [NA]	NA	NA	NA	NA	NA	NA	NA	32% [16-53]

NA = not available; AOFAS = American Orthopaedic Foot and Ankle Score; VAS = Visual Analogue Scale; NRS = Numeric Rating Scale; FAOS = Foot and Ankle Outcome Score; ADL = activities of daily living; QoL = quality of life; EQ-5D = EuroQol; AAS = Ankle Activity Scale; RTW = return to work; RTS = return to sports (values are presented in means); CI = confidence interval.

### Sports and Work Outcomes

Sports outcomes were assessed in 4 of the 5 studies.^19,26-28^ Return to sport time was only reported by Reilingh *et al*.^27^ and was calculated to be 19 weeks (range 5-49 weeks). Return to any level of sports rate was 83% across the 4 studies (95% CI, 70-91). In 2 studies,^19,26^ return to pre-injury level of sports rate was reported, and the mean RTS to pre-injury level rate was assessed to be 55% (95% CI, 34-74). Return to work was reported in 3 studies,^26-28^ and the mean rate of return to work was 92% (95% CI, 78-97). Return to work time was reported in 2 studies,^26,27^ and the mean return to work time was 7 weeks (range, 2-14).

### Imaging Assessment

Imaging outcomes were reported in 3 studies.^26-28^ Two studies^26,28^ reported postoperative degenerative osteoarthritic changes, while Reilingh *et al*.^27^ reported CT (computed tomography) scans in 11 patients at 1-year follow-up in whom the subchondral bone plate quality (level and filling) and filling of the defect was assessed. In the study of Ogilvie-Harris DJ, Sarrosa,^26^ there were no osteoarthritic changes as assessed by the Kellgren and Lawrence scale at final follow-up. In the study of Schuman *et al*.^28^ 6% of patients developed degenerative changes at a follow-up 10 years after the surgery as assessed with postoperative radiographs. The level of subchondral bone plate was depressed in 91% of the patients in the study by Reilingh *et al*.,^27^ while it was flush in 9% of the patients. Concerning subchondral bone plate filling, 55% of the patients showed incomplete filling, while 45% of the patients showed fully complete or almost fully complete filling of the subchondral bone plate. Filling of the defect was reported to be between 0% and 33%, 34% and 66%, and 67% and 100% of the initial volume in 18%, 9%, and 73% of the patients, respectively.

### Complications and Revision Surgery

For 3 studies,^26,27,29^ it was possible to extract data on complications as the other 2 studies either did not report on complications or it was not possible to extract separate data on complication occurrence. The complication rate was 10% (95% CI, 4-22) with 4 complications reported (1 temporary hypoesthesia of the dorsum of the foot in the first webspace, 1 paraesthesia of the foot, 1 delayed wound healing, and 1 persistent deep ankle pain after a novel distortion at 4-month follow-up). Revision or reoperation surgery was reported in 4 studies.^19,27-29^ The revision rate was calculated to be 27% (95% CI, 18-40), with 1 HemiCap implantation, 2 reoperations of unknown nature, and 14 osteochondral autograft transplantations.

## Discussion

The most important finding of the present study is that the overall success rate of arthroscopic BMS for nonprimary OLTs was low at 61% (95% CI, 50-72), and the re-revision rate was high. Furthermore, return to sports and work outcomes yielded fair to good results. To the best of our knowledge, this is the first systematic review investigating the clinical outcomes of nonprimary BMS for failed primary talar osteochondral lesions.

This study demonstrated that 61% of the patients showed successful clinical outcomes following BMS for secondary OLT, collecting data from 5 different studies. When comparing this to a recent systematic review^1^ on solely primary lesions, this percentage is relatively low. Dahmen *et al*.^1^ found that the clinical success rate for primary lesions was 82% with a 95% confidence interval ranging from 78 to 86% with mean follow-up time of 38 months (ranging from 10 to 143 months). A potential explanation for this lower percentage may be that patients who did not respond to a primary BMS procedure as an index procedure may not respond well to a secondary procedure, potentially due to inherently poor biology particularly. Furthermore, in most lesions following microfracture the lesion size increased after the index procedure secondary to poor bone and cartilage healing. This finding is supported by clinical evidence,^10-12^ as larger lesions do not respond well to BMS.^8^ Another potential explanation of the lower clinical success percentage may be the unknown individual patient factors, such as stem cell characteristics, and may as well be the longer follow-up time in the present study versus the previous study performed by Dahmen *et al*.^1^ on solely primary lesions (49 months vs. 38 months, respectively). However, this difference may be considered marginal, so large differences in clinical success rates may not be expected. Moreover, the success rate of 61% may be considered low due to the influence of the inclusion of the study by Yoon *et al*.^29^; this study had the lowest success rates of all the included studies with the highest number of patients included (*n* = 22, success rate = 32% [95% CI, 16-53]). A potential explanation for this calculated success rate may be the fact that the authors included a high percentage of cystic (64%) and uncontained (86%) lesions; both cystic and uncontained lesions have been previously found to be negatively associated with clinical outcomes after BMS.^6,17,30^

Yoon *et al*.^29^ stated in their study that worse outcomes were observed in larger lesions separating lesions smaller and larger than 150 mm^2^. The authors found that patients with small defects demonstrated clinical failure in 53% of the patients with small lesions, versus 100% of the patients with large lesions. Another explanation for this finding may be that the subchondral bone plate may have resulted in inferior quality.^13,31-35^ This theory is supported by the findings of Reilingh *et al*.^27^ in the present study showing that 91% of the secondary lesions showed a depressed subchondral bone plate. Multiple studies have shown that the subchondral bone plate and the subchondral bone itself play a vital role in overall cartilage health and repair.^36-38^ A recent study by Shimozono *et al*.^39^ showed that after BMS the subchondral bone was not restored at midterm follow-up when assessing 42 patients. Moreover, the authors observed a significant decrease of overall subchondral bone health scores over time and found that these subchondral bone health scores were positively corrected with clinical outcomes. It is therefore hypothesized that impairments to the subchondral bone may irreversibly alter the joint-loading support, resulting in fibrocartilage degradation over time and increased wear and tear of the joint.^13,31^

As an opposing explanation for the fact that 6 out of 10 patients responded clinically well to the secondary BMS procedure, it may be hypothesized that the lesions had not been fully debrided during the index procedure, potentially because of inferior visualization of the defect during the arthroscopic index procedure. However, Savva *et al*.^19^ found that in their study population this may not have been the case as otherwise a more dramatic subsequent increase in lesion size was to be expected at the secondary arthroscopic BMS. Additionally, they found that at repeat arthroscopy the lesions that were operated on showed low-quality detached fibrocartilage. This finding is in line with other studies on second-look arthroscopic findings, such as the study by Lee *et al*.^10^ in which the authors showed that 40% of the lesions had incompletely healed with fibrocartilage. Moreover, Yang *et al*.^40^ recently concluded that 36% of the patients were incompletely healed and had inferior quality of repair tissue in comparison to native cartilage at a mean follow-up of 3.6 years. While our study found that 61% of the patients had successful outcomes after secondary BMS, it must be stated that the study with the longest follow-up time, the study by Yoon *et al*.,^29^ showed the lowest individual success rate: 32% (95% CI, 16-53). This is an important clinical topic of interest, as approximately 4 out of 10 patients will demonstrate progression of osteoarthritis of the ankle at longer follow-up times.^9,12^

When assessing the sports outcomes in the present review, it was found that 83% of the patients returned to any level of sport and 55% of the patients were able to return to a pre-injury level of sport. Comparing these analyses to the findings of a recent systematic review by Steman *et al*.,^41^ return to any level of sports is comparable; 83% for solely nonprimary lesions after repeat arthroscopic BMS, versus 88% for mostly primary lesions. However, the return to pre-injury level of sports rate for the secondary lesions in our analyses showed that 55% of the patients were able to return to this level, while another study^41^ revealed that 79% of the patients were able to return after arthroscopic BMS. The possible differences for these discrepancies in rates of return to sport are not yet fully understood.

The clinical relevance of this systematic review is that the pooled outcomes of secondary BMS for nonprimary OLT can be used to inform patients about which outcomes are to be expected concerning clinical success rates, sports and work outcomes, revision and complication, as well as radiological outcomes. This may ameliorate the shared decision-making process between patients and physicians, making the decision between a repeat arthroscopy or a form of (osteo)chondral transplantation for the individual patient more evidence based.

### Limitations

The present study has to be interpreted in light of its strengths and limitations. First, it can be observed that the majority of the studies were of low methodological quality, except for the study of Reilingh *et al*.,^27^ which can be regarded a high-quality publication in the form of an RCT. Additionally, one can appreciate that the mean defect size was below 150 mm^2^. This finding, in combination with a lack of comparative (prospective) studies reporting the differences between repeat arthroscopic BMS versus a different treatment strategy within the same surgical indication (namely, size of lesion, patients’ complaints, and lesion morphology), made it impossible to perform a formal meta-analysis utilizing mixed-effects logistic regression analyses in order to compare between treatment groups, which can consequently be regarded as a limitation of the study. Another limitation is that the included studies used subjective scoring systems, such as the AOFAS score, which are not officially validated for the clinical evaluation of the treatment of OLTs; future studies should focus on developing validated outcome measures for the treatment of OLTs. The strengths of this review are the inclusion of solely nonprimary lesions having undergone BMS, thereby purely focusing on a highly selected group of lesion types and patients which yields novel insights into the clinical effectiveness of this surgical intervention. Other strengths that need to be mentioned are the extensive corresponding author contact protocol, the thorough reference selection, and the quality assessment of the included studies.

## Conclusion

The overall success rate of arthroscopic BMS for nonprimary OLT was low at 61% and was accompanied by a high revision rate of 27%. Return to sports and work outcomes yielded fair to good results.

## Appendix

**Table:**

PubMed	
#	Searches
1	“Osteochondritis Dissecans”[Mesh]
2	Osteochondritis dissecans[tiab] OR osteochondrosis dissecans[tiab] OR osteochondrolysis[tiab] OR OCD[tiab] OR OLT[tiab]
3	(osteochondral[tiab] OR chondral[tiab] OR transchondral[tiab] OR cartilage*[tiab]) AND (defect*[tiab] OR lesion*[tiab])
4	#1 OR #2 OR #3
5	“Talus”[Mesh]
6	talus[tiab] OR talar*[tiab] OR ankle[tiab]
7	#5 OR #6
8	#4 AND #7
EMBASE (Ovid)	
#	Searches
1	(osteochondritis dissecans/ or (osteochondritis dissecans or osteochondrosis dissecans or osteochondrolysis or OCD or OLT).ti,ab,kw. or ((osteochondral or chondral or osteochondral or transchondral or cartilage*) adj3 (defect* or lesion*)).ti,ab,kw.) and (talus/ or (talus or talar* or ankle).ti,ab,kw.)
Cochrane Library	
#	Searches
1	MeSH descriptor: [Osteochondritis Dissecans] explode all trees
2	(osteochondritis dissecans or osteochondrosis dissecans or osteochondrolysis or OCD or OLT):ti,ab,kw (Word variations have been searched)
3	((osteochondral or chondral or transchondral or cartilage*) and (defect* or lesion*)):ti,ab,kw (Word variations have been searched)
4	#1 or #2 or #3
5	MeSH descriptor: [Talus] explode all trees 34
6	(talus or talar* or ankle):ti,ab,kw (Word variations have been searched)
7	#5 or #6
